# Carbon dioxide fluxes from contrasting ecosystems in the Sudanian Savanna in West Africa

**DOI:** 10.1186/s13021-014-0011-4

**Published:** 2015-01-13

**Authors:** Emmanuel Quansah, Matthias Mauder, Ahmed A Balogun, Leonard K Amekudzi, Luitpold Hingerl, Jan Bliefernicht, Harald Kunstmann

**Affiliations:** 1 Federal University of Technology, Akure, Nigeria; 2 Institute of Meteorology and Climate Research, Karlsruhe Institute of Technology, Garmisch-Partenkirchen, Germany; 3 Kwame Nkrumah University of Science and Technology, Kumasi, Ghana; 4 Chair for Regional Climate and Hydrology, University of Augsburg, Augsburg, Germany; 5 Head of Chair for Regional Climate and Hydrology, University of Augsburg, Augsburg, Germany

**Keywords:** West Africa, Sudanian Savanna, Carbon fluxes, Net ecosystem exchange

## Abstract

**Background:**

The terrestrial land surface in West Africa is made up of several types of savanna ecosystems differing in land use changes which modulate gas exchanges between their vegetation and the overlying atmosphere. This study compares diurnal and seasonal estimates of CO_2_ fluxes from three contrasting ecosystems, a grassland, a mixture of fallow and cropland, and nature reserve in the Sudanian Savanna and relate them to water availability and land use characteristics.

**Results:**

Over the study period, and for the three study sites, low soil moisture availability, high vapour pressure deficit and low ecosystem respiration were prevalent during the dry season (November to March), but the contrary occurred during the rainy season (May to October). Carbon uptake predominantly took place in the rainy season, while net carbon efflux occurred in the dry season as well as the dry to wet and wet to dry transition periods (AM and ND) respectively. Carbon uptake decreased in the order of the nature reserve, a mixture of fallow and cropland, and grassland. Only the nature reserve ecosystem at the Nazinga Park served as a net sink of CO_2_, mostly by virtue of a several times larger carbon uptake and ecosystem water use efficiency during the rainy season than at the other sites. These differences were influenced by albedo, LAI, EWUE, PPFD and climatology during the period of study.

**Conclusion:**

These results suggest that land use characteristics affect plant physiological processes that lead to flux exchanges over the Sudanian Savanna ecosystems. It affects the diurnal, seasonal and annual changes in NEE and its composite signals, GPP and RE. GPP and NEE were generally related as NEE scaled with photosynthesis with higher CO_2_ assimilation leading to higher GPP. However, CO_2_ effluxes over the study period suggest that besides biomass regrowth, other processes, most likely from the soil might have also contributed to the enhancement of ecosystem respiration.

## Background

Globally, the terrestrial biosphere has been recognised to have the capacity to partially offset anthropogenic CO_2_ by carbon assimilation into the biomass through the process of photosynthesis [1,2]. The African ecosystems contribute significantly to the inter-annual variability in the global atmospheric CO_2_, mainly through the emission of trace gases from land use changes and bush burning [3,4]. The terrestrial ecosystem in West Africa is made up of several types of savannas which have exhibited strong diurnal and seasonal variability in their CO_2_ fluxes, an indication of how the carbon budget can be affected by the ecosystem’s response to meteorological forcing [5]. For example, Brümmer et al. [6] showed that a Southern Sudanian Savanna ecosystem in Burkina Faso was a source of CO_2_ into the atmosphere in the dry season, but only marginally, whereas appreciable CO_2_ uptake was observed in the rainy season. In addition, Ardö et al. [7] found that for a sparse savanna in the semi-arid Sudan, during two short investigation periods, in February 2005 (dry season), and September 2005 (rainy season), the studied ecosystem was a sink of carbon during both periods.

The contributions of the West African savanna ecosystems to the global carbon budgets as well as factors influencing their impact on the temporal and spatial variation of the terrestrial carbon uptake and emission are still highly uncertain. An insufficient network of sites using measurement equipment to determine CO_2_ fluxes such as eddy covariance (EC) stations have been identified as one of the main reasons [6]. Although over the years many EC experiments have been performed in West Africa within various research projects such as the Sahelian Energy Balance EXperiment (SEBEX, [8]), Hydrological and Atmospheric Pilot Experiment-Sahel in Niger (HAPEX-Sahel, [9-11]), the African Monsoon Multidisciplinary Analyses (AMMA) project [12], and the CARBOAFRICA project [13], the network of eddy covariance stations in West Africa is still very sparse compared to the network in North America, Europe and Asia, [14]. A situation which has resulted in a limited scope of comprehensive climatic information essential for the development of regional climate adaptation policies especially in setting CO_2_ reduction targets. Therefore, attempts to generalise or extrapolate outcomes of flux measurements from one area to another area of a given target region based on results from former flux measurements [15] are associated with high uncertainties. The main reason is that carbon dynamics vary for different ecosystems, depending on the climate, land management and further factors. Another reason is that many former measurement experiments were mostly performed for short periods, e.g. for a specific season or for several months and for a few selected sites [15,16].

As part of the efforts to develop effective adaptation and mitigation measures to climate change and land use in West Africa, the meteorological network has been refined in three regions of the Sudanian Savanna of West Africa by installing further climate stations and three eddy covariance (EC) stations. The establishment of the EC and climate stations has been realised within the framework of the West African Science Service Centre on Climate Change and Adapted Land Use (WASCAL) project. The EC stations have been established in October 2012 and January 2013 in the West African Sudanian Savanna along a gradient of changing land use (cover) characteristics (grassland, a mixture of fallow and cropland and nature reserve), close to the border between Ghana and Burkina Faso [17].

The objective of this study was to perform an inter-comparison of CO_2_ flux exchanges across three contrasting ecosystems, and to quantify their magnitude and temporal variability in net ecosystem exchange (NEE), gross primary production (GPP) and ecosystem respiration (ER) in responses to changing vegetation characteristics and different soil moisture conditions. This will improve our understanding of the impact of physiological and environmental factors on CO_2_ efflux and sequestration patterns over the region. It will also contribute to our knowledge regarding how NEE and its composite signals (GPP and ER) vary with land use change.

## Results and discussions

### General meteorology

The meteorological conditions (air temperature and relative humidity) over the study areas were evaluated at each individual EC site. The results (Figure not shown) revealed that the variability in the daily averages of air temperature (Tair), relative humidity (RH) and precipitation during the study period was typical of the Sudanian Savanna. The highest daily mean Tair of 34.49, 34.73 and 34.88°C, were recorded in March in the dry season (November – April) for the Nazinga Park, Sumbrungu and Kayoro respectively. While the minimum daily mean Tair of 22.04, 22.58 and 23.27°C occurred in August for Kayoro and in September, the rainy season (May–October) for the Nazinga Park and Sumbrungu respectively. The daily averaged RH ranged between 8.64 to 94.38%, 7.86 to 93.28 and 7.80 to 95.13% for the Nazinga Park, Sumbrungu and Kayoro respectively. The minimum values of RH were recorded in February, while the maximum values were obtained in August for the Nazinga Park and Kayoro, and in September for Sumbrungu.

**Figure 1:**
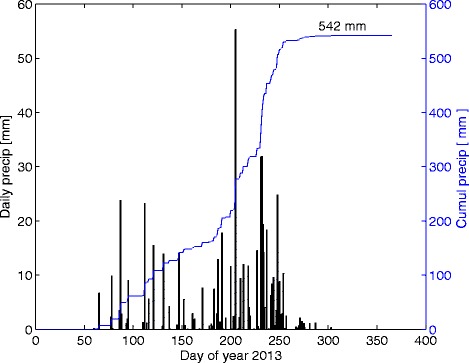
**Daily sums of precipitation from a climate station at Bongo Soe in the Upper East region of Ghana.** Also included is the cumulative sum over the study period.

Annual rainfall obtained from Sumbrungu was approximately 375 mm yr^-1^. In this station, gaps (N = 188) in the dry season (December) were assumed to be zero. Results from a ‘gap-free’ data at a nearby climate station in Bongo Soe (Table 1) with similar vegetation characteristics as Sumbrungu was 542 mm yr^-1^ (Figure 1). This suggest rainfall over the region was sporadic during the period. Nevertheless, our values were still within what had been reported (320–1100 mm) over the Southern Sudanian Savanna [7,18-21]. Annual rainfall from the remaining EC stations could not be determined because of several gaps in the rainfall data, especial during the rainy season.

**Table 1:** **Coordinates of the climate stations surrounding the three eddy covariance sites**

**Name of climate station**	**Latitude (** ^**o**^ **)**	**Longitude (** ^**o**^ **)**	**Elevation (m)**
**Bongo Soe**	10.973	-0.783	200
Doninga	10.617	-1.420	162
Nabuobelle	10.703	-1.869	328
Oualem	11.205	-1.309	295
Nebou	11.305	-1.879	310
Tabou	11.367	-2.169	312
Gwasi	10.478	-1.648	284

### Volumetric soil moisture content

**Figure 2:**
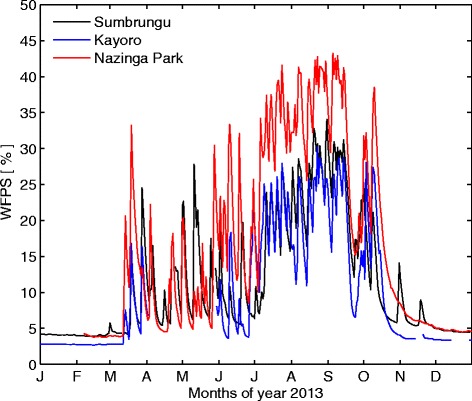
**Daily mean of soil moisture content, converted into water filled pores space at the three eddy covariance sites.**

The daily averages of the volumetric water content measured from the topsoil at 0.03 m from each EC sites were converted into water-filled pore space (WFPS) for the one-year study period based on the formula described in Brümmer et al. [6]. The results during the period ranged between 3.90 to 34.05% for Sumbrungu, 2.69 to 30.24% for Kayoro and 3.76 to 43.25% for the station in the Nazinga (Figure 2), which suggest that the top few centimetres of the soil (5 cm) was not entirely dried (not below 3%) even in the dry season (November to April). The soil moisture increased after the onset of the rain in March in the dry season. Afterwards, the soil water content decreased drastically again until the beginning of the rainy season in May. There was strong variability in the WFPS during the rainy season (May to October), but no clear indication of the soil moisture reaching saturation condition. The soil moisture begun to dry up at the end of the rainy season in October. However, the WFPS was never close to zero. There was a strong correlation between the near surface soil moisture and the precipitation, an indication of a quick response to rain events by the topsoil.

### Evaluation of the energy balance closure

**Figure 3:**
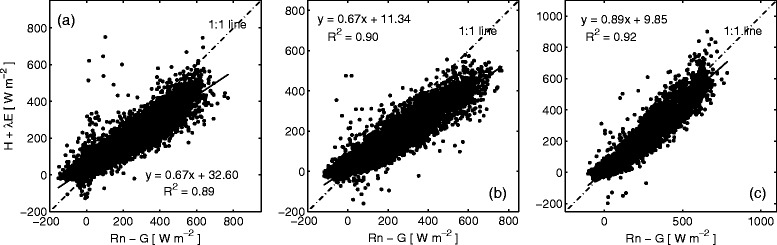
**Energy balance closure for the study period at (a) Sumbrungu, (b) Kayoro, all in the Upper West Region of Ghana, and (c) the Nazinga Park near Pô in the Nahouri province in Burkina Faso.**

The half-hourly averaged flux and meteorological data set of G, H, λE and R_n_ (number of data points (N) = 12240, 11470 and 10291) for Sumbrungu, Kayoro and Nazinga respectively, were used to evaluate the performance of the eddy covariance measurements for the entire study period, based on the linear regression relation between the turbulent fluxes (H + λE) and available energy (R_n_ – G). The regression fits between (R_n_ − G) and (H + λE) resulted in a coefficient of determination of 0.89 for Sumbrungu, 0.90 for Kayoro and 0.92 for Nazinga. The slopes and intercepts were 0.67 and 32.60 W m^-2^, 0.67 and 11.34 W m^-2^, and 0.89 and 9.85 W m^-2^ for the three sites accordingly (Figure 3). The slopes of our regression indicated that the energy balance closures (EBC) were moderate for Sumbrungu and Kayoro. Our results were within the typical closure since the sum of the turbulence fluxes (H + λE) is usually found to be 10 to 30% less than the available energy (Rn – G) due to large-scale transport not captured by regular eddy covariance tower measurements and measurement errors associated with individual instruments [22-24].

The EC station at Kayoro was selected to determine the impact of meteorological conditions on the EBC for four different periods within the year (JFM, AM, JJASO and ND), that corresponded with the complete dry season, dry to wet transition, rainy season, and wet to dry transition respectively. The results revealed that the EBC was variable with slopes of 0.66, 0.50, 0.65 and 0.58 according to the analysed periods. In addition, analyses of the EBC at Kayoro for two selected days, one without rain and the other with total rain of 8.6 mm day^-1^ in the rainy season, gave a slight reduction in the EBC for the latter. These suggested that over the studied period the EBC could have been affected by the changes in the meteorological conditions prevalent during the dry and rainy seasons. It is therefore likely that besides the possible causes of the non-closure of the EBC as mentioned above, the scattered feature of the points observed over the one-year study period was due to the sensitivity of the measurement EC device to rain drops and dust. As we focused on the carbon dioxide fluxes, no adjustments were made to compensate for the imbalances in the EBC in this study, which is in accordance with Foken et al. [25].

### Seasonal changes in daily NEE

**Figure 4:**
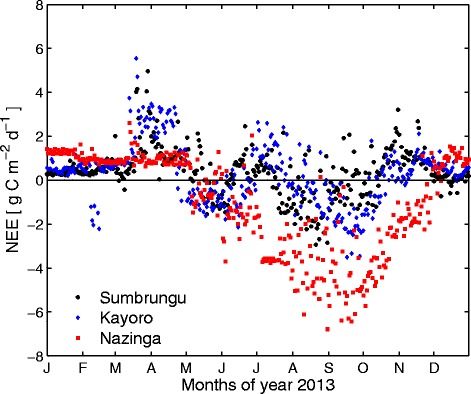
**Seasonal changes in daily sums of net ecosystem exchange at the eddy covariance sites.**

The seasonal changes in daily sums of NEE for the three study sites are illustrated in Figure 4. There were effluxes between March and April, the transition between the dry to rainy season. And these were prominent for Sumbrungu and Kayoro, peaking at 4.97, 5.55 and 2.62 g C m^-2^ d^-1^ for Sumbrungu, Kayoro and Nazinga respectively. This was followed predominantly by net uptake for Nazinga between May and peaking at -6.78 g C m^-2^ d^-1^ at the end of August, and decreasing until the end of November, the transition period between the rainy and dry season. However for Sumbrungu and Kayoro both uptakes and effluxes were observed between the rainy season (May to October), with maximum uptakes in August and September at -2.93 and -3.51 g C m^-2^ d^-1^ for Sumbrungu and Kayoro respectively.

The occurrence of the maximum daily NEE uptake in the rainy season over the one-year investigated period, revealed the important roles precipitation and soil moisture play on the rates of CO_2_ uptakes and effluxes at the studied ecosystems.

**Figure 5:**
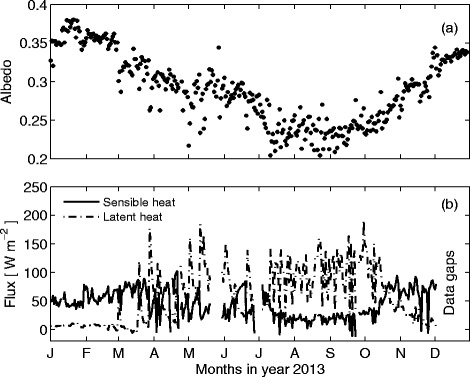
**Seasonal changes in daily mean of (a) albedo, and (b) energy fluxes (sensible and latent heat) at Sumbrungu.**

The evolution of the albedo, a function comprising of the surface and radiation characteristics, such as the land cover type, vegetation phenology, soil moisture, incident angle, and wavelength [26-28], was assessed at the study sites over the period. The results suggested a decrease in albedo from the onset of the rain in March until the end of November, before it started to rise again until the end of December. Figure 5 shows the results of the daily mean albedo and the daily mean energy fluxes (sensible and latent heat) during the period for Sumbrungu, which ranged between 0.20–0.38. Similar patterns were observed for Kayoro and Nazinga (Figures not shown), between 0.16–0.29 and 0.12–0.27 respectively. The high albedo values were observed in the dry season when most of the leaves on the ecosystem had withered and sensible heat dominated the energy exchanges. The lower values occurred in the rainy season when an enhancement in soil moisture (high latent heat) led to productivity pulsation, followed by the accumulation of biomass. Leaf area index measured for Sumbrungu and Kayoro between June and October 2013 ranged between 1.3–2.5 and 3.0–5.3 m^2^ m^-2^ respectively.

**Figure 6:**
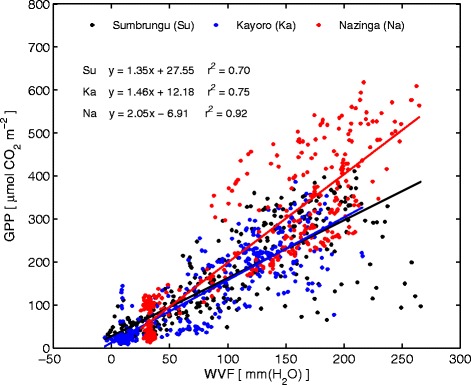
**Daily sums of gross primary production against daily sums of water vapour flux.**

The ecosystem water use efficiency (EWUE) was simply calculated as the ratio of the rate of net CO_2_ uptake (GPP) to the water vapour flux (WVF) according to Law et al. [29]. However, no filtering was applied to remove overestimation as a result of precipitation events as described in Grelle et al. [30]. The linear regression between the daily integrated GPP and WVF (Figure 6), revealed that the slopes (EWUE) were 1.35, 1.46 and 2.05 μmol CO_2_ m^-2^ mm(H_2_O)^-1^ for Sumbrungu, Kayoro and the Nazinga Park respectively, during the entire period of the study. This suggest evapotranspiration and gross primary production were strongly related and that EWUE showed daily variability throughout the year. However, the values of EWUE obtained for the three sites during the rainy season (May to October) were 1.68, 1.71 and 2.17 μmol CO_2_ m^-2^ mm(H_2_O)^-1^ for Kayoro, Sumbrungu and Nazinga Park respectively. This was an indication that the difference in the climatology between the dry and rainy season as well as the land surface characteristics and the morphology of the vegetation influenced the NEE and its composite fluxes.

**Figure 7:**
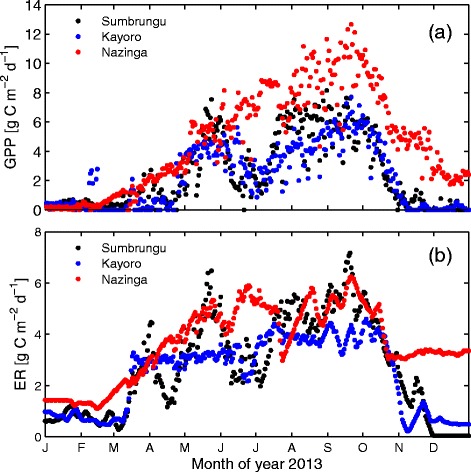
**Seasonal changes in (a) daily sums of gross primary production, and (b) daily sums of ecosystem respiration.**

**Figure 8:**
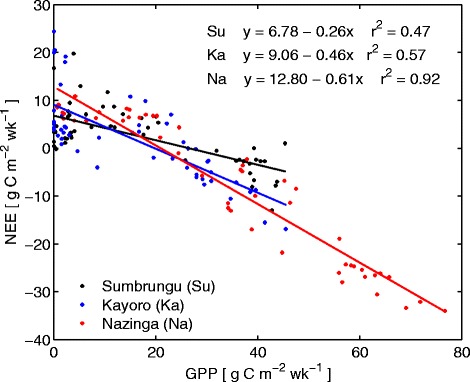
**Scaling of weekly sums of ecosystem carbon exchange (NEE = net ecosystem exchange) with weekly sums of ecosystem photosynthesis (GPP = gross primary production).**

**Figure 9:**
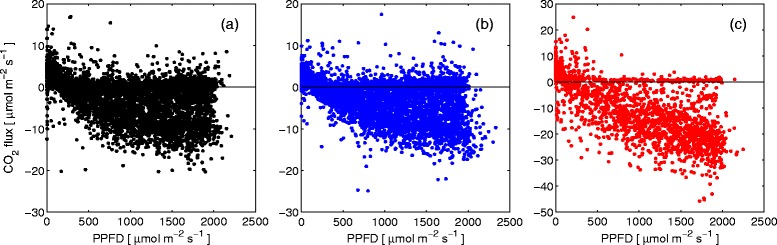
**Half-hourly values of net ecosystem exchange in relation to half-hourly values of photosynthetic photon flux density for (a) Sumbrungu, (b) Kayoro and (c) the Nazinga Park.** Positive values are net release of CO_2_ into the atmosphere, while negative values represent net uptake by the ecosystem.

Increase in soil moisture during the rainy season, enhanced the processes leading to both autotrophic and heterotrophic respiration, which resulted in increasing rates in the daily sums of GPP and ER in the increasing order, from Kayoro to Sumbrungu and to Nazinga (Figure 7), which agreed well with the EWUE values obtained in the rainy season for the respective stations. Figure 8 illustrates the disparities in how the NEE scaled with photosynthesis. Higher carbon dioxide assimilations (GPP) coincided with higher uptake rates of NEE decreasing in the order of Nazinga, Kayoro and Sumbrungu accordingly. The higher values of NEE during the day for all the three sites coupled with photosynthetic photon flux density (PPFD, Figure 9), with maximum NEE occurring at PPFD of 2000 μmol m^-2^ s^-1^.

### Seasonal variability of monthly carbon fluxes

**Figure 10:**
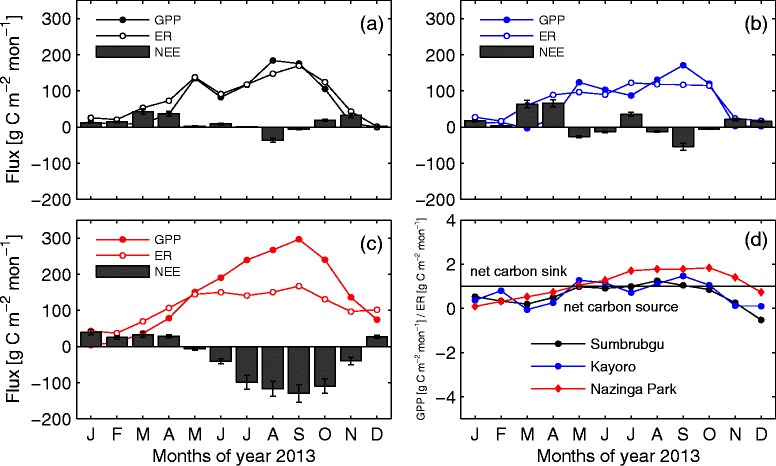
**Seasonality of monthly NEE, GPP, ER at (a) Sumbrungu, (b) Kayoro, (c) the Nazinga and (d) ratio of the monthly sums of GPP to the monthly sums of ER at the three study sites.**

The seasonality of the carbon fluxes including the GPP and ER derived from the half-hour measurements of the gap-filled-NEE at the study areas was investigated using their monthly sums (Figure 10). The outcomes illustrate strong variability in the monthly fluxes for the three sites over the study period, mostly influenced by the soil moisture. The NEE for the complete dry season (JFM) for Sumbrungu, Kayoro and Nazinga were respectively, 68.58, 83.25 and 97.52 g C m^-2^. In contrast, during the rainy season (JJASO) NEE values were -14.29, -51.02 and -494.68 g C m^-2^ for Sumbrungu, Kayoro and Nazinga respectively. Analyses for the transition periods, i.e. the dry to wet transition (AM) showed NEE rates at 39.56, 39.35 and 21.90 g C m^-2^ for Sumbrungu, Kayoro and Nazinga. While the wet to dry transition (ND), also showed NEE rates at 1.96, 15.46 and 27.32 g C m^-2^. The results suggest that the ecosystems served as sources of CO_2_ during the dry season (November – April), as well as the transition periods AM and ND when soil moisture was low. However, during the rainy season (May–October), the ecosystems served as sinks of CO_2_ at all the three sites. During this period the NEE in Kayoro was more than three times that at Sumbrungu, while that of Nazinga was almost ten times that of Kayoro. While all three ecosystems had a similar NEE during the dry season, the nature reserve ecosystem was a much larger carbon sink in the rainy season. Furthermore, the results for ND revealed a quick switch from net uptake to net release during the year, but the same cannot be said for AM.

In July 2013, during one of the routine visits to the field site in Kayoro, it was observed that a residence in a nearby community had planted cowpea (*Vigna unguiculata*) in the vicinity of the EC setup that extended into our fetch. Although we removed the portion that affected our fetch to allow for regrowth of grass, it is likely that the preparations for the cowpea plantation caused the sudden release of CO_2_ in July (Figure 10b). If this is the case, then considering the fact that cowpea is one of the principal crops cultivated in the region, land management over the region could have considerable impact on the diurnal, seasonal and annual variability of CO_2_ uptake and release across the region.

The outcomes of the monthly (seasonal) integrated total amount of productivity in the ecosystem, GPP and ER derived from the gap-filled NEE over the period for all the study sites showed that, in all cases, the assimilation of carbon (GPP) was mostly limited to the rainy season when soil moisture content was increased. Furthermore, ER rates were clearly higher in the rainy season compared to the complete dry season as well as the transitions periods (Figure 10a,b and c). This may be attributed to the enhancement of the processes that lead to both autotrophic and heterotrophic respirations ‘fuelled’ by the general plant growth driving forces (precipitation and soil moisture). The higher carbon assimilation (GPP) rates corresponded with higher NEE uptakes in the rainy season. The GPP to ER flux partitioning ratios (Figure 10d) revealed a net carbon uptake (GPP/ER > 1) for months from May to November and peaking in October, the end of the rainy season, especially for Nazinga. The ratios decreased after November as soil water content declined (see Figure 2) until the beginning of the rainy season in May. In the dry season, high air temperature and low relative humidity values led to a high vapour pressure deficit. This resulted in a stronger driving forces on evapotranspiration and hence low ER rates during the complete dry season (JFM) and the transition periods (AM and ND), compared to the rainy season.

**Figure 11:**
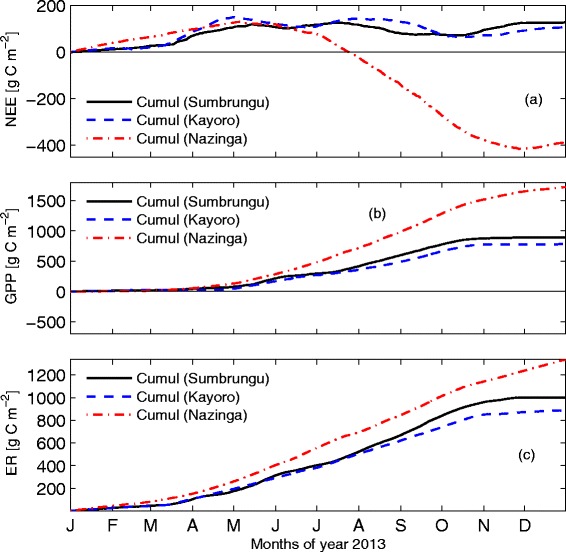
**Annual aggregation of (a) measured NEE, (b) estimated GPP, and (c) estimated ER over the study sites.**

The estimated annual (January to December) carbon fluxes (NEE, GPP and ER) derived from their cumulative daily sums over the period showed that only the nature reserve ecosystem at Nazinga served as a net sink of CO_2_ at -387.3 ± 23.1 g C m^-2^ yr^-1^, while the remaining two sites were net sources of CO_2_ into the atmosphere at 127.8 ± 7.2 and 108.0 ± 5.5 g C m^-2^ yr^-1^ for Sumbrungu and Kayoro respectively (Figure 11a). The corresponding values of the annual cumulative GPP and RE over the period (Figures 11b and c) are provided in Table 2.

**Table 2:** **Summary of the seasonal and annual integrated carbon fluxes (NEE = net ecosystem exchange, GPP = gross primary production, ER = respiration; [g C m** ^**-2**^ **(time unit)** ^**-1**^ **]) over the study sites, with their corresponding standard deviations (std)**

**Variable**	**Sumbrungu**		**Kayoro**		**Nazinga Park**	
	**Value**	**std**	**Value**	**std**	**Value**	**std**
**Rainy season NEE**	-11.8	0.5	-77.6	4.6	-501.2	38.9
Dry season NEE	139.5	10.8	185.6	12.3	113.9	6.4
Rainy season GPP	798.0	108.3	734.4	95.5	1385.0	193.4
Dry season GPP	76.0	11.8	46.9	6.4	340.6	57.1
Rainy season RE	786.3	102.8	656.8	87.1	883.4	114.0
Dry season RE	215.5	34.2	232.5	36.7	454.4	65.8
Annual NEE	127.8	7.2	108.0	5.5	-387.3	23.1
Annual GPP	874.0	17.8	781.3	15.8	1725.1	32.6
Annual ER	1001.8	19.0	889.3	16.5	1337.8	23.0

Comparison of our GPP results with the alternative flux partitioning approach of Lasslop et al. [31] resulted in coefficients of determinations of 0.74, 0.89 and 0.92 and regression slopes of 0.80, 0.92 and 0.96 for Nazinga, Kayoro and Sumbrungu respectively. Similarly, the ER comparison gave coefficient of determination of 0.36, 0.79 and 0.83 and regression slopes of 0.65, 0.97 and 0.70 for Nazinga, Kayoro and Sumbrungu respectively. While the NEE comparison resulted in slopes of 0.84, 0.70 and 0.70 and coefficient of determination of 0.84, 0.71 and 0.67 for Nazinga, Kayoro and Sumbrungu respectively. This relatively good agreement shows that the MDS gap-filling technique is quite suitable for our study sites within the typical uncertainty range of such estimates of 20 to 30% [32]. Although the MDS approach resulted in larger estimates of both NEE, GPP and ER, the estimate for the cumulative NEE agreed quite well for all three sites (Table 2).

### Comparison with selected former flux measurements

Our daily values of photosynthetically driven carbon uptake of -2.93, -3.51 and -6.78 g C m^-1^ d^-1^ for the grassland, mixture of fallow and cropland and nature reserve respectively compared well with values of -0.48, -1.8, -2.4 -3.6 and -5.9 g C m^-1^ d^-1^ obtained for some former flux measurements (Table 3). Similarly, the rates of daily release of CO_2_ from our study sites at 4.97, 5.55 and 2.62 g C m^-2^ d^-1^ for Sumbrungu, Kayoro and Nazinga respectively, were comparable with values of 0.4, 0.6 and 2.4 g C m^-1^ s^-1^, from the former flux measurements (Table 3) [7]. The monthly and annual fluxes also compared well. Although the comparison revealed some slight variability in CO_2_ efflux and uptake values for the variety of ecosystems presented (Table 3), the general observation revealed that soil moisture as well as other physiological properties of the ecosystems were very significant in the CO_2_ sequestration and efflux patterns over the regions. Example, a nature reserve near Bontioli in Burkina Faso, which shared similar characteristics with our third EC site, the Nazinga Park, produced total net ecosystem CO_2_ uptake of -179 ± 98 g C m^-2^ yr^-1^ in the first year and -429 ± 100 g C m^-2^ yr^-1^ in the second year of investigations. And the large difference in values between the two years was attributed to the increment in the rainfall amount, 785 mm, in the first year as against 919 mm in the second year [6]. Comparing those daily, monthly and annual estimates with values obtained from our study sites suggest that ecosystems that shared similar characteristics were more comparable with each other.

**Table 3:** **Net ecosystem exchange from former flux measurements**

**Site**	**Vegetation**	**MAP**	**Annual NEE**	**Monthly NEE**	**Daily NEE**
Baja	Desert shrub	174	-39, -52	0.7 – 25^D^	0.4^D^
				-12 to -41^R^	-0.48^R^
Demokeya	Sparse savanna	320	-	-	-0.2^D^
					-1.8^R^
Maun	Savanna woodland	464	12	-	1.2 - 2.4^D^
					-0.6 to -2.4^R^
Hapex	Sahelian fallow savanna	495	-32	-	~0.3^D^
					0.6 to -3.6^R^
Virgina Park	Open woodland savanna	667	44	~6^D^	-
				14 to -52^R^	-
Bontioli	Shrub dominated savanna	926	-179, -429	5 -20^D^	0.2 – 0.4^D^
				-35 to -175^R^	-1.4 to -5.9^R^
Aguas Emendadas	Cerrado	1500	-		0.6^D^
				-	-1.2^R^
Sumbrungu	Short grassland savanna	375	127.8	2 - 43^D^	4.97^D^
				-6 to -37^R^	-2.93^R^
Kayoro	Fallow land	-	108	3 - 66^D^	5.55^D^
				-6 to -54^R^	-3.51^R^
Nazinga Park	Nature reserve	-	-387.3	25 - 39^D^	2.62^D^
				-6 to -130^R^	-6.78^R^

[NEE] = g C m^-2^ (time unit)^-1^.

MAP – Mean Annual Precipitation, mm yr^-1^.

NEE – negative values denote uptake.

^R^ – Rainy season.

^D^ – Dry season.

The annually estimated GPP values obtained from our studies were 874.0, 781.3 and 1725.1 g C m^-2^ yr^-1^ (Table 2). These looked similar to values obtained from studies summarised in Sjöström et al. [33] for a variety of ecosystems in Africa that ranged approximately between 50 to 2050 g C m^-2^ yr^-1^.

## Summary and conclusions

The carbon fluxes of three contrasting ecosystem in the Sudanian Savanna in West Africa have been estimated using one year of eddy covariance CO_2_ flux data. Three eddy covariance stations were built close to the Ghanaian-Burkinabe border in October 2012 and January 2013. The first EC site represents a grassland ecosystem, which served as a pasture occasionally to the cattle and sheep owned by the inhabitants living in the nearby communities. The natural vegetation in the area is characterised by grasses of average height, 0.10 m when fully grown after the rainy season in October. The second site is dominated by tall grasses with an average height of 1 m after the rainy season in October and represents a mixture of fallow and cropland, and the third EC site is a representation of a near-natural Sudanian Savanna with no agricultural activities. The latter is located in a nature reserve area and characterised by tall grass of approximately 3 m when fully grown after the rainy season in October.

Carbon uptake predominantly took place during the rainy season, while a net carbon release was observed in the dry season as well as during the transition periods between the dry and rainy seasons (i.e. AM and ND). The seasonal trends in NEE varied substantially among all the three sites. Climatology and biophysical factors including, LAI, albedo, surface roughness length modulated the phenological processes leading to biomass regrowth and CO_2_ assimilation at the study sites. It appeared that soil moisture availability played a significant role in the variability of NEE during the transition periods.

Our study revealed that land use and management had a large impact on the disparities in NEE values at the study sites. Generally, the carbon uptake decreased in the order from nature reserve over mixture of fallow and cropland to grassland. Only the nature reserve ecosystem at Nazinga served as a net sink of CO_2_, mostly by virtue of a much larger carbon uptake and ecosystem water use efficiency during the rainy season than at the other sites.

In order to improve our knowledge about the impact of the plant-physiological processes on flux exchanges over the three contrasting ecosystems, numerical simulations of energy and CO_2_ fluxes as well as a gap-filling model with a special parameterisation adapted to such rain driven ecosystems over the Sudanian Savanna are warranted.

## Materials and methods

### Study site description

**Figure 12:**
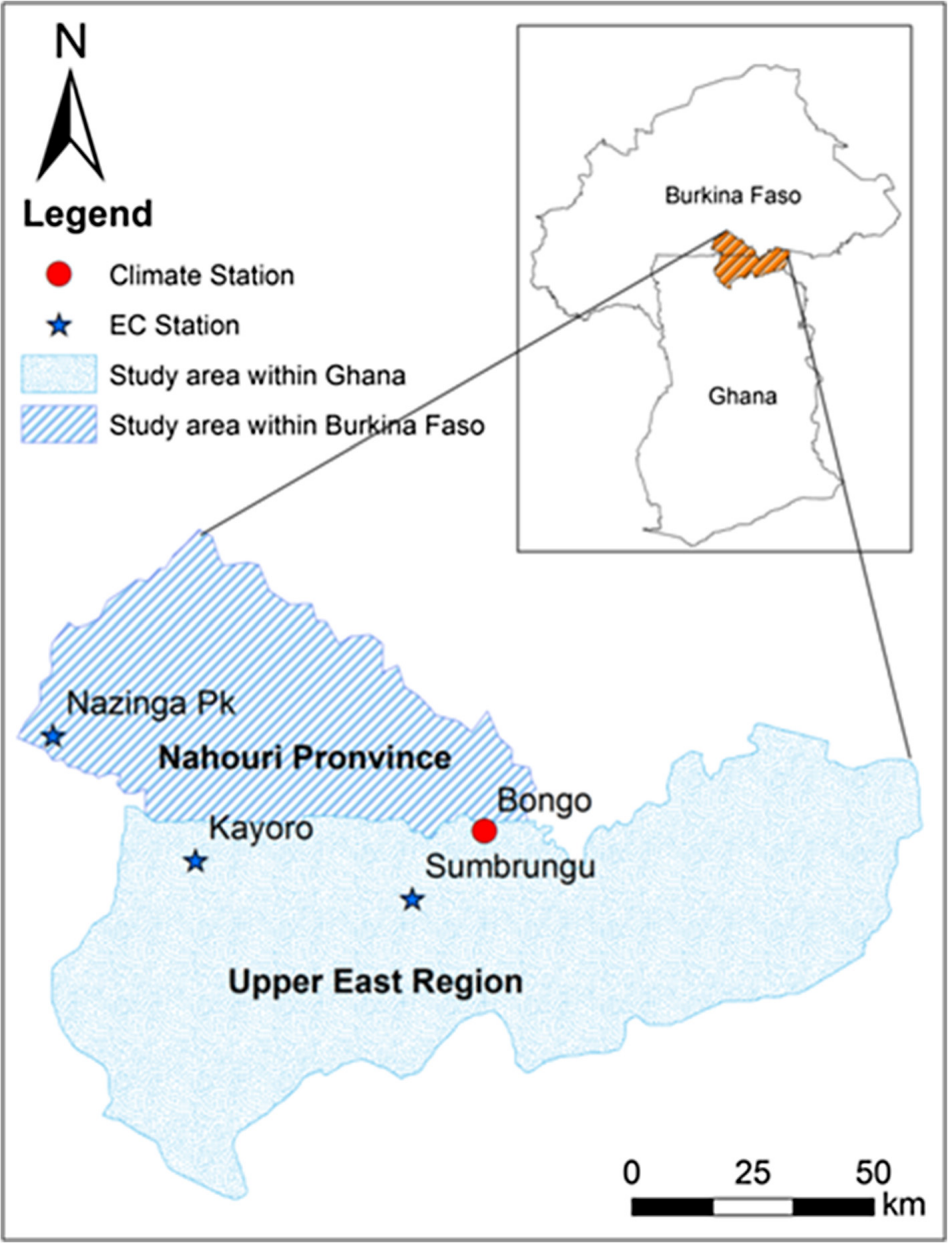
**EC sites located in the Sudanian Savanna belt in the Upper East Region of Ghana and the Nahouri province of Southern Burkina Faso.** In addition, locations of the climate stations are indicated.

Three eddy covariance stations were built close to the Ghanaian-Burkinabe border (Figure 12) in October 2012 and January 2013. The first EC station was located in Sumbrugu Aguusi (10.846^o^ N, 0.917^o^ W, 200 m a.s.l.) within the catchment of the river Vea, about 35 km from Bolgatanga in the Upper East Region of Ghana. This site was representative of a grassland ecosystem which occasionally served as pasture for some livestock (e.g. cattle and sheep) owned by inhabitants of the nearby communities. The stocking density of the grazing animals ranged between 6–8 animals per hectare. The natural vegetation in the area was characterised by grasses (e.g. *Brachiaria lata, Chloris piloasa and Cassia mimosoides*) of average height, 0.10 m when fully grown after the peak of the rainy season in October. The leaf area index (LAI) measurements undertaking between June and October 2013 gave values ranging between 1.3–2.5 m^2^ m^-2^. The area was interspersed with trees of heights between 3–5 m and around 30–40 m apart. *Parkia biglobosa, Adansonia* and *Lannea microcarpa* were some of the dominant tree species in this area. The measurements were carried out in an area dominated by grass. The location of the eddy covariance system was selected in a manner to allow for the required fetch. The tower position was chosen in order that all the trees were at a distance of more than 30 m away and were not within the two main wind directions (easterly and westerly) during the study period. The micrometeorological instruments were installed in a 2 m high wired-fence surrounding an area of 7 m × 7 m to protect the devices from destruction by the livestock.

The second EC station was established at Kayoro Dakorenia (10.918^o^ N, 1.321^o^ W, 292 m a.s.l.) within the catchment of the Tono river, approximately 100 km away from Bolgatanga in the Upper East Region of Ghana. The vegetation of this site was dominated by tall grasses (e.g. *Andropogon and Cenchrus*) with an average height of 1 m after the rainy season in October. The LAI measured between June and October 2013 were between 3.0–5.3 m^2^ m^-2^. The dominant tree species in the area included: *Entada africana, Acacia dudgeoni and Vitellaria paradoxa*. This site represented a mixture of fallow and cropland, occasionally used for cropping. Similarly at this site, the tower location allowed for sampling from grass dominated areas and all the trees were at distances of more than 200 m away and were also not within the dominant wind directions during the period of the study.

The third eddy covariance station was located in the Nazinga Park (11.152^o^ N, 1.586^o^ W, 293 m a.s.l.), one of the biggest natural reserves in Burkina Faso, about 50 km from Pô in the Nahouri province (Figure 12). It was located in the Sissili river basin. The site represented a near-natural Sudanian Savanna with no agricultural activities. It was located at a specific research area of the natural reserve close to the Nazinga Ranch, and endowed with rivers with many small dams close to the ranch with rich biological diversity (mammals, birds, and flora). The eddy covariance instrument was located in a protected area of the nature reserve reserved for research purposes. The vegetation of the site was made up of a mosaic of shrubs and tree savannas (e.g. *Daniellia oliveri, Burkea Africana and Isoberlinia doka*), between 4–5 m tall with averaged canopy height of about 4.5 m. While the grasses were mainly of annual and perennial Graminae, with average height of about 2.5 m at the end of the rainy season in October. The LAI was not determined for this site.

### Climate of the study areas

All the three study sites are representative of the Sudanian savanna climate and vegetation, with a mono-modal rainfall pattern, mainly between the months of May and October, while the dry season begins from November and ends in April each year [34,35]. The mean annual precipitation is between 320 and 1100 mm [7,18-21]. The general climate is characterised by the seasonal changes in water availability and fires which are influenced by the dry and rainy seasons, also linked to the movement of the Inter-Tropical Convergence Zone (ITCZ, [36]).

The dry season is associated with dry and dust-laden ‘Harmattan’ winds with low relative humidity and low night temperatures, while the contrary prevails during the rainy season. The horizontal wind characteristics are predominantly north easterly in the dry season, but south westerly in the rainy season. The observed temperatures over the area were low during the month of August with an average of 22°C but the high values were recorded between March and April, with a peak value of about 40°C, while the annual relative humidity were between 6 to 95% [15,37,38].

### Instrumentation

All three EC stations were equipped with the same measurement devices. The EC sensors included CSAT3 3D sonic anemometer (Campbell Scientific Inc., USA) to measure wind speed and direction as well as the sonic temperature, and a Licor 7500A open-path infrared gas analyser (LI-COR, Biosciences Inc., USA) to measure CO_2_ and H_2_O in the atmosphere. In addition, each station was equipped with a tipping bucket, model 52293 (R. M. Young, USA) and a weighing gauge pluviometer (Ott, Germany) to measure rainfall, the HMP155A (Campbell Scientific Inc., USA) to measure air temperature and relative humidity and the CNR4 net radiometer (Kipp & Zonen, Netherlands) to measure the incoming and outgoing shortwave and longwave radiations. Furthermore, the CS616 soil moisture probes (Campbell Scientific Inc., USA) were employed to measure the volumetric soil moisture content, at the depth of 0.03 m at each site, while the TCAV thermal sensors (Campbell Scientific Inc., USA), and the HFP01SC self-calibrating heat flux plates (Hukseflux, Netherlands) were used to measure soil temperature at three different depths (0.03, 0.10, and 0.30 m) and soil heat fluxes at 0.08 m respectively. Also installed were MX-Q24M-Sec-D11 web cameras (Mobotix, Germany) to take daily pictures from the surrounding areas for studying the vegetation phenology. In addition, the multi-sensor WXT 520 (Vaisala, Finland) was installed to measure rainfall, air temperature, horizontal wind speed and direction, relative humidity and air pressure. Data from each station were automated and recorded onto the CR3000 and CR1000 data loggers (Campbell Scientific Inc., USA). Two solar panels were built at each station for the power supply of the EC stations and the data transmission unit for an automatic transfer of the measurements on the daily basis.

### Determination of Carbon dioxide, water vapour and energy fluxes

The carbon dioxide and the energy flux data were recorded at the three study sites at high-frequency of 20 Hz. The flux and meteorological data from each study site were stored every 30 minutes. The turbulence (vertical) fluxes of the carbon dioxide, *F*_*c*_ (mmol m^-2^ s^-1^), the sensible heat flux, *H* (W m^-2^) and the latent heat flux, *λE* (W m^-2^) for each time step of the investigated period were calculated as described in [39,40]:

1 $$ {\mathrm{F}}_{\mathrm{c}} = {\uprho}_{\mathrm{a}}\overline{\mathrm{w}\mathit{\hbox{'}}\uprho {\mathit{\hbox{'}}}_{\mathrm{c}}} $$

2 $$ \mathrm{H} = {\uprho}_{\mathrm{a}}{\mathrm{c}}_{\mathrm{p}}\overline{\mathrm{w}\mathit{\hbox{'}}\kern0.5em \mathrm{T}{\mathit{\hbox{'}}}_{\mathrm{a}}} $$

3 $$ \uplambda \mathrm{E} = \uplambda \overline{\mathrm{w}\mathit{\hbox{'}}\uprho {\mathit{\hbox{'}}}_{\mathrm{v}}} $$

where *ρ*_*a*_ is the density of dry air (kg m^-3^) at a given air temperature, *c*_*p*_ is the specific heat capacity of dry air at constant pressure (J kg^-1^ K^-1^), *λ* is the latent heat of vapourisation (J kg^-1^), *ρ*_*c*_ is the molar density of CO_2_ gas (mol m^-3^) and *ρ*_*v*_ is the molar density of water vapour (mol m^-3^). *T*_*a*_ is the air temperature derived from the sonic anemometer (K) and *w* is the vertical wind velocity component (m s^-1^). Over bars denote time averages and primes indicate fluctuations about the averages.

### Measurement and parameterisation of ground heat flux

The soil heat flux is often assumed to be negligible in many studies. The heat fluxes are often estimated from in situ soil measurements which are affected by measurement errors. This can lead to large uncertainties regarding the estimation of the heat fluxes [41], which also strongly influences the energy balance closure.

In this paper, the soil heat fluxes were measured at each station using three heat flux plates, each buried at 0.08 m depth and 0.05 m apart within the soil. The average heat fluxes from these plates at each time intervals (30 minutes) were used as the measured soil heat fluxes. However, the change of the heat storage (*G*_*s*_) above the plates at 0.03 m depth was calculated based on a simplified equation proposed by Liebethal et al. [41], which uses the measured soil temperature at 0.03 m at each time step. Hence the soil or ground heat flux was calculated based on the combination method, the sum of the measured heat flux and the calculated heat storage (*G*_*s*_):

4 $$ \mathrm{G}={\mathrm{G}}_{\mathrm{m}}+{\mathrm{G}}_{\mathrm{s}} $$

where *G* is the ground heat flux (W m^-2^), *G*_*m*_ is the measured ground heat flux (W m^-2^) using the heat flux plates and *G*_*s*_ is the calculated heat storage (W m^-2^) above the heat flux plates. *G*_*s*_ was calculated based on the following formula:

5 $$ {\mathrm{G}}_{\mathrm{s}}={\displaystyle {\int}_0^{\mathrm{z}}{\mathrm{C}}_{\mathrm{v}}\frac{\partial {\mathrm{T}}_{\mathrm{s}}}{\partial \mathrm{t}}\mathrm{d}\mathrm{z}} $$

where *z* is the depth (m) above the heat flux plate from the soil surface, *T*_*s*_ is the soil temperature (°C) measurements at 0.03 m at each time step (30 minutes) and *t* is the time intervals (s). *C*_*v*_, the volumetric heat capacity (J m^-3^ K^-1^), was estimated from the composition of the soil following De Vries [42]:

6 $$ {\mathrm{C}}_{\mathrm{v}} = \left(1.90\cdotp {\mathrm{f}}_{\mathrm{m}}+2.47\cdotp {\mathrm{f}}_{\mathrm{o}}+4.12\cdotp {\mathrm{V}}_{\mathrm{s}}\right)\cdotp {10}^6 $$

where *f*_*m*_ is the volumetric fraction of minerals (%) in the soil, *f*_*o*_ is the fraction of organic content in the soil (%) and *V*_*s*_ is the time dependent (30 minute) volumetric soil moisture content (m^3^ m^-3^). The values of *f*_*m*_ and *f*_*o*_ (see Table 4) were kept constant throughout the investigated period.

**Table 4:** **Summary of the characteristics of the eddy covariance sites, including the measurement height of the open-path gas analyser and selected soil physio-chemical properties taking from soil samples at 0.10 m**

	**Sumbrungu**	**Kayoro**	**Nazinga Park**
Location (Lat/Lon)	10.846^o^ N	10.918^o^ N	11.152° N
	0.917^o^ W	1.321^o^ W	1.586° W
Elevation (m)	200.00	292.00	293.00
Management	Highly degraded used for grazing	Mixture of fallow and cropland	Nature reserve
EC height (m)	2.65	3.15	7.19
Average height of grass when fully grown after the rainy season (m)	0.10	1.00	3.00
Average canopy height of trees (m)	4.50	3.00	4.50
Land cover type	Short grass savanna	Tall grass savanna	Tall grass/shrub savanna
Soil texture	Loamy sandy	Loamy sandy	Sandy loam
Soil bulk density (g cm^-3^)	1.41	1.42	1.42
Organic carbon (%)	1.60	0.62	3.30
Soil mineral (%)	51.74	52.84	50.30
C:N ratio	11.07	11.00	11.28
pH	5.79	6.13	6.37
Sand (%)	77.99	77.09	51.52
Silt (%)	16.34	20.05	38.93
Clay (%)	5.67	2.87	9.53

### Data acquisition and quality control

The CO_2_ and the energy flux data were obtained from the three study sites using the eddy covariance method [43,44]. The raw turbulence data including the mixing ratios of CO_2_, H_2_O, air temperature and pressure, as well as the three-dimensional wind speeds were processed using the software TK3.1, which had the capability to perform all post field processing of turbulence measurements and to produce statistically quality assured turbulence fluxes for a station automatically [40]. Within the TK3.1 software, a number of calculations and standard corrections for open-path sensors to produce quality controlled (*QC*) and quality assured (*QA*) data were performed after the raw data such as air temperature, water vapour, CO_2_ and the three components of the wind velocity (*u, v* and *w*) had been retrieved from the CR3000 and CR1000 data loggers. The programme applied standardised quality assessment routines and user specified consistency limits [40,45,46], to detect and reject physically or electronically not possible values of the EC data including CO_2_, sensible heat (*H*) and the latent heat (*λE*) fluxes, emanating from unfavourable micrometeorological conditions, such as strong non-stationary and non-turbulence as well as low-quality data caused by precipitation, dust, or other contamination on the sensor optics. The study state quality test of the mean and variance of the data set at 20 Hz scanning frequency and 5 minutes interval was performed based on Foken et al. [47]. The spikes were detected according to Vickers and Mahrt [48], based on Højstrup [49], and these were values which exceeded 4.5 times standard deviations in a window of 15 values. However, in a situation where this criterion was fulfilled by 4 or more values in a row, they were not considered as such. They were supposed to be ‘real’ in this case. The Planar Fit method after Wilczak et al. [50] was applied for the co-ordinate transformation. Moreover, the Schotanus et al. [51] correction was applied for obtaining the sensible heat flux from the sonic measurements and the Webb et al. [52] correction was applied to account for density fluctuations in the fluxes of carbon dioxide and water vapour.

All the climatic and eddy covariance variables used in this work, except the gross primary production and ecosystem respiration were determined using the aforementioned eddy covariance setups, meteorological instruments and equations.

### Energy balance closure

The performance of the eddy covariance measurements was evaluated based on the linear regression relation between the half-hourly averaged turbulent fluxes (*H + λE*) and the available energy (*R*_*n*_ *– G*):

7 $$ \mathrm{H}+\uplambda \mathrm{E}=\mathrm{m}\left({\mathrm{R}}_{\mathrm{n}}-\mathrm{G}\right)+\mathrm{b} $$

where *m* is the slope and *b* (W m^-2^) is the intercept respectively. Under an ideal condition the *m* should be equal to 1. However, the sum of the turbulence fluxes is usually found to be between 10 to 30% less than the available energy due to several factors such as large-scale transport not captured by regular eddy covariance tower measurements and measurement errors associated with individual instruments [23,45,53].

### Data gap-filling and flux partitioning schemes

The flux of CO_2_ across the interface between the vegetation and the atmosphere, the net ecosystem exchange (NEE), at the study sites can directly be calculated via the eddy covariance approach. The sign convention of NEE is with respect to the atmosphere, i.e. a negative sign means the ecosystem captures CO_2_ from the atmosphere, while a positive sign means the ecosystem is losing CO_2_ to the atmosphere. The NEE is comprised of signals made up of photosynthetic uptake, gross primary production (GPP), as well as autotrophic and heterotrophic respiration, called the ecosystem respiration (ER). The gaps in NEE data that emanated from strong non-stationary and non-turbulence, power failures, spikes, precipitation and dust among others, accounted for 33, 39 and 43% for Sumbrungu, Kayoro and the Nazinga Park respectively, during the study periods. These gaps were consistent with data gaps of between 30 to 40%, usually found in flux measurements on an annual time scale which were required to be gap-filled [2,54,55], in order to obtained continuous data for the calculations of the daily, monthly (seasonal) or annual integrated carbon balances.

In this paper, the NEE was gap-filled as well as decomposed into its constituent signals (GPP and RE) using the online gap filling and flux partitioning tool publicly available at: (http://www.bgc-jena.mpg.de/~MDIwork/eddyproc/: [56]. This Marginal Distribution Sampling (MDS) technique has a similar performance compared to other techniques [2]. The gap filling process using the MDS technique requires meteorological data (30 minutes averages) such as solar radiations, air temperature, relative humidity, vapour pressure deficit and the soil temperature. The technique employs temporal auto-correlation of fluxes, and estimate the missing data based on the mean value of available data under similar meteorological conditions within an averaging window of 7 days. In the case of larger data gaps with no similar meteorological conditions within the 7-day window, the averaging window is increased by 14 days until similar meteorological conditions are met. The flux partitioning component of the on line tool estimates the ecosystem respiration (ER) by employing the regression model proposed by Lloyd and Taylor [57]:

8 $$ \mathrm{E}\mathrm{R}\left({\mathrm{T}}_{\mathrm{s}}\right)={\mathrm{E}\mathrm{R}}_{\mathrm{ref}}.{\mathrm{e}}^{{\mathrm{E}}_{\mathrm{o}}\left(\frac{1}{{\mathrm{T}}_{\mathrm{ref}}-{\mathrm{T}}_{\mathrm{o}}}-\frac{1}{{\mathrm{T}}_{\mathrm{s}}-{\mathrm{T}}_{\mathrm{o}}}\right)} $$

where *T*_*s*_ (^o^C) is the soil temperature, *T*_*o*_ (°C) is the regression parameter, *E*_*o*_ (K^-1^) is the activation-energy that determines temperature sensitivity, *T*_*ref*_ (°C) is the reference temperature and *ER*_*ref*_ (μmol m^2^ s^-1^) is the ecosystem respiration at the reference temperature. The regression parameter *E*_*o*_ and the reference temperature *T*_*ref*_ were kept constant as in the original model by Lloyd and Taylor [57]. GPP was finally derived from the difference between NEE and ER. In order for easy comparison of our results with those from the former flux measurements, e.g. Brümmer et al. [6], Ardö et al. [7] and others, the daily, monthly (seasonal) and annual courses of the carbon fluxes (NEE, GPP and ER) were expressed as g C m^-2^ (time unit)^-1^. Results of former measurements in other units were also converted into g C m^-2^ (time unit)^-1^ for easy comparability.

## Abbreviations

AM: April-May
AMMA: African Monsoon Multidisciplinary Analyses
BMBF: German Federal Ministry of Education and Research
CARBOAFRICA: Project to quantify, understand and predict carbon cycleand other greenhouse gases in Sub-Saharan Africa
EBC: Energy balance closure
EC: Eddy covariance
ER: Ecosystem respiration
EWUE: Ecosystem water use efficiency
GPP: Gross primary production
HAPEX-Sahel: Hydrological and Atmospheric Pilot Experiment-Sahel
ITCZ: Inter-Tropical Convergence Zone
JFM: January-February-March
JJASO: June-July-August-September-October
LAI: Leaf area index
MDS: Marginal Distribution Sampling
NEE: Net ecosystem exchange
ND: November-December
PPFD: Photosynthetic photon flux density
QA: Quality assurance
QC: Quality control
RH: Relative humidity
SEBEX: Sahelian Energy Balance Experiment
Tair: Air temperature
TK3.1: ‘Turbulentzknecht’ version 3.1 (software for processing of eddy fluxes)
VPD: Vapour pressure deficit
WASCAL: West African Science Service Centre on Climate Change and Adapted Land Use
WFPS: Water-filled pore space
WVF: Water vapour flux
ZEF: Centre for development research

## References

[1] Janssens IA, Freibauer A, Ciais P, Smith P, Nabuurs G-J, Folberth G (2003). Europe’s terrestrial biosphere absorbs 7 to 12% of European anthropogenic CO_2_ emissions. Science.

[2] Thomas MV, Malhi Y, Fenn KM, Fisher JB, Morecroft MD, Lloyd CR (2010). Carbon dioxide fluxes over an ancient broadleaved deciduous woodland in southern England. Biogeosciences Discuss.

[3] Bombelli A, Valentini R (2011). Africa and Carbon Cycle. World Soil Resources Reports No. 105.

[4] Ciais P, Bombelli A, Williams M, Piao SL, Chave J, Ryan CM (2011). The carbon balance of Africa: synthesis of recent research studies. Phil Trans R Soc.

[5] Pilegaard K, Hummelshoej P, Jensen NO, Chen Z (2001). Two years of continuous CO_2_ eddy-flux measurements over a Danish beech forest. Agric Forest Meteorol.

[6] Brümmer C, Falk U, Papen H, Szarzynski J, Wassmann R, Brüggemann N. Diurnal, seasonal, and interannual variation in carbon dioxide and energy exchange in shrub savanna in Burkina Faso (West Africa). J Geophys Res. 2008;113:G02030. doi:10.1029/2007JG000583.

[7] Ardö J, Mölder M, El-Tahir BA, Elkhidir HAM (2008). Seasonal variation of carbon fluxes in a sparse savanna in semi-arid Sudan. Carbon Balance Manage.

[8] Wallace JS, Wright IR, Steward JB, Holwill CJ (1991). The Sahelian Energy Balance Experiment (SEBEX): Ground based measurements and their potential for spatial extrapolation using satellite data. Adv Space Res.

[9] Verhoef A, Allen S, De Bruin H, Jacobs C, Heusinkveld B (1996). Fluxes of carbon dioxide and water vapour from a Sahelian savanna. Agric For Meteorol.

[10] Friborg T, Boegh E, Soegaard H (1997). Carbon dioxide flux, transpiration and light response of millet in the Sahel. J Hydrol.

[11] Hanan NP, Kabat P, Dolman JA, Elbers JA (1998). Photosynthesis and carbon balance of a Sahelian fallow savanna. Glob Chang Biol.

[12] Redelsperger J-L, Thorncroft CD, Diedhiou A, Lebel T, Parker DJ, Polcher J (2006). African monsoon multidisciplinary analysis: an international research project and field campaign. Bull Am Meteorol Soc.

[13] Bombelli A, Henry M, Castaldi S, Adu-Bredu S, Arneth A, de Grandcourt A (2009). An outlook on the Sub-Saharan Africa carbon balance. Biogeosciences.

[14] A Global Network – Historical Site Status [http://fluxnet.ornl.gov/site_status; 13-5-2014]

[15] Bagayoko F (2006). Impact of land-use intensity on evaporation and surface runoff: Processes and parameters for eastern Burkina Faso, West Africa.

[16] Schüttemeyer D. The Surface Energy Balance Over Drying Semi-Arid Terrain in West Africa, PhD thesis. University of Wageningen, Netherlands; 2005.

[17] Bliefernicht J, Kunstmann H, Hingerl L, Rummler T, Andresen S, Mauder M, Steinbrecher R, Frieß R, Gochis D, Gessner U, Quansah E, Awotuse A, Neidl F, Jahn C, Barry B (2013). Field and simulation experiments for investigating regional land-atmosphere interactions in West Africa: experimental set-up and first results.

[18] Kpongor D. Spatially Explicit Modeling of Sorghum (Sorghum bicolor L) Production on Complex Terrain of a Semi‐arid Region of Ghana using APSIM, PhD thesis. University of Bonn, Germany; 2007.

[19] Brümmer C, Papen H, Wassmann R, Brüggemann N. Fluxes of CH_4_ and CO_2_ from soil and termite mounds in south Sudanian savanna of Burkina Faso (West Africa). Global Biogeochem Cycles. 2009;23:GB1001. doi:10.1029/2008GB003237.

[20] Grote R, Lehmann E, Brümmer C, Brüggemann N, Szarzynski J, Kunstmann H (2009). Modelling and observation of biosphere atmosphere interactions in natural savannah in Burkina Faso, West Africa. Phys Chem Earth.

[21] Ibrahim B, Polcher J, Karambiri H, Rockel B (2012). Characterization of the rainy season in Burkina Faso and it’s representation by regional climate models. Clim Dyn.

[22] Foken T (2008). The energy balance closure problem - An overview. Ecolog Appl.

[23] Ingwersena J, Steffens K, Högy P, Warrach-Sagi K, Zhunusbayeva D, Poltoradnev M (2011). Comparison of Noah simulations with eddy covariance and soil water measurements at a winter wheat stand. Agric For Meteorol.

[24] Leuning R, Eva van G, Massman WJ, Isaac PR (2012). Reflections on the surface energy imbalance problem. Agric For Meteorol.

[25] Foken T, Leuning R, Oncley SP, Mauder M, Aubinet M, Aubinet M, Vesala T, Papale D (2012). Corrections and data quality. Eddy Covariance: A Practical Guide to Measurement and Data Analysis.

[26] Henderson-Sellers A, Wilson MF (1983). Albedo observations of the Earth's surface for climate research. Phil Trans Roy Soc London.

[27] Wenge N, Woodcock C (1999). “Surface albedo of boreal conifer forests: Modeling and measurements,” Geoscience and Remote Sensing Symposium, IGARSS '99 Proceedings. IEEE 1999 International.

[28] Schmitt M, Bahn M, Wohlfahrt G, Tappeiner U, Cernusca A (2010). Land use affects the net ecosystem CO_2_ exchange and its components in mountain grasslands. Biogeosciences.

[29] Law BE, Falge E, Gu L, Baldocchi DD, Bakwin P, Berbigier P (2002). Environmental controls over carbon dioxide and water vapor exchange of terrestrial vegetation. Agr Forest Meteorol.

[30] Grelle A, Lundberg A, Lindroth A, Moren A-S, Cienciala E (1997). Evaporation components of a boreal forest: variations during the growing season. J Hydrol.

[31] Lasslop G, Reichstein M, Papale D, Richardson AD, Arneth A, Barr AG (2010). Separation of net ecosystem exchange into assimilation and respiration using a light response curve approach: critical issues and global evaluation. Glob Chang Biol.

[32] Schulze ED, Ciais P, Luyssaert S, Schrumpf M, Janssens IA, Thiruchittampalam B (2010). The European carbon balance. Part 4: integration of carbon and other trace-gas fluxes. Glob Chang Biol.

[33] Sjöström M, Zhao M, Archibald S, Arneth A, Cappelaere B, Falk U (2013). Evaluation of MODIS gross primary productivity for Africa using eddy covariance data. Remote Sens Environ.

[34] Ofori-Sarpong E (2001). Impact of climate change on agriculture and farmers coping strategies in the upper east region of Ghana. West Afr J Appl Ecol.

[35] Callo-Concha D, Gaiser T, Ewert F. Farming and cropping systems in the West African Sudanian Savanna. WASCAL research area: Northern Ghana, Southwest Burkina Faso and Northern Benin, ZEF Working Paper Series, No. 100, 2012. Available at: http://hdl.handle.net/10419/88290.

[36] Sultan B, Janicot S (2003). The West African monsoon dynamics, Part II: The “pre-onset” and the “onset” of the summer monsoon. J Clim.

[37] Oguntunde PG. Evapotranspiration and Complimentarity Relations in the Water Balance of the Volta Basin: Field Measurements and GIS-based regional Estimate. In: Vlek PLG, Denich M, Martius C, van de Giesen N, editors. Ecology and Development Series, vol. 22. Cuvillier Verlag, Göttingen: 2004. p. 1–170.

[38] Sandwidi JP. Groundwater Potential to Supply Population Demand within the Kompienga dam basin in Burkina Faso. In: Vlek PLG, Denich M, Martius C, Rodgers C, van de Giesen N, editors. Ecology and Development Series, vol. 55. Cuvillier Verlag, Göttingen: 2007. p. 1–157.

[39] Grünwald T, Bernhofer C (2007). A decade of carbon, water and energy flux measurements of an old spruce forest at the Anchor Station Tharandt. Tellus.

[40] Mauder M, Foken T (2011). Documentation and Instruction Manual of the Eddy-Covariance software Package TK3.

[41] Liebethal C, Huwe B, Foken T (2005). Sensitivity analysis for two ground heat flux calculation approaches. Agric For Meteorol.

[42] De Vries DA. Thermal Properties of Soils. In Physics of Plant Environment. Edited by VanWijk WR. Amsterdam: North-Holland Publishing Company; 1963:210 – 235.

[43] Kaimal JC, Finnigan JJ (1994). Atmospheric Boundary Layer Flows: Their Structure and Measurement.

[44] Aubinet M, Grelle A, Ibrom A, Rannik Ü, Moncrieff J, Foken T (2000). Estimates of the annual net carbon and water exchange of European forests: the EUROFLUX methodology. Adv Ecol Res.

[45] Mauder M, Jegede OO, Okogbue EC, Wimmer F, Foken T (2007). Surface energy balance measurements at a tropical site in West Africa during the transition from dry to wet season. Theor Appl Climatol.

[46] Mauder M, Cuntz M, Drüe C, Graf A, Rebmann C, Schmid HP (2013). A strategy for quality and uncertainty assessment of long-term eddy-covariance measurements. Agric For Meteorol.

[47] Foken T, Wichura B (1996). Tools for quality assessment of surface-based flux measurements. Agric For Meteorol.

[48] Vickers D, Mahrt L (1997). Quality control and flux sampling problems for tower and aircraft data. J Atmos Ocean Technol.

[49] Højstrup J (1993). A statistical data screening procedure. Meas Sci Technol.

[50] Wilczak JM, Oncley SP, Stage SA (2001). Sonic anemometer tilt correction algorithms. Bound-Layer Meteor.

[51] Schotanus P, Nieuwstadt FTM, De Bruin HAR (1983). Temperature measurement with a sonic anemometer and its application to heat and moisture fluxes. Bound-Layer Meteorol.

[52] Webb EK, Pearman GI, Leuning R (1980). Correction of the flux measurements for density effects due to heat and water vapour transfer. Quart J Roy Meteorol Soc.

[53] Stoy PC, Mauder M, Foken T, Marcolla B, Boegh E, Ibrom A (2013). A data-driven analysis of energy balance closure across FLUXNET research sites: The role of landscape scale heterogeneity. Agric For Meteorol.

[54] Moffat AM, Papale D, Reichstein M, Hollinger DY, Richardson AD, Barr AG (2007). Comprehensive comparison of gap-filling techniques for eddy covariance net carbon fluxes. Agric For Meteorol.

[55] Baldocchi D (2008). TURNER REVIEW No. 15, “Breathing” of the terrestrial biosphere: lessons learned from a global network of carbon dioxide flux measurement systems. Aust J Bot.

[56] Reichstein M, Falge E, Baldocchi D, Papale D, Valentini R, Aubinet M (2005). On the separation of net ecosystem exchange into assimilation and ecosystem respiration: review and improved algorithm. Global Change Biol.

[57] Lloyd J, Taylor JA (1994). On the temperature dependence of soil respiration. Funct Ecol.

